# The Use of β-Blockers in Heart Failure with Reduced Ejection Fraction

**DOI:** 10.3390/jcdd8090101

**Published:** 2021-08-24

**Authors:** Daniele Masarone, Maria Luigia Martucci, Vittoria Errigo, Giuseppe Pacileo

**Affiliations:** Heart Failure Unit, Department of Cardiology, A. O. dei Colli, Monaldi Hospital, 80131 Naples, Italy; marilumartucci93@gmail.com (M.L.M.); vittoria.errigo@gmail.com (V.E.); gpacileo58@gmail.com (G.P.)

**Keywords:** β-blockers, heart failure with reduced ejection fraction, pharmacologic therapy

## Abstract

Treatment with β-blockers is the main strategy for managing patients with heart failure and reduced ejection fraction because of their ability to reverse the neurohumoral effects of the sympathetic nervous system, with consequent prognostic and symptomatic benefits. However, to date, they are underused, mainly because of the misconception that hypotension and bradycardia may worsen the haemodynamic status of patients with HFrEF and because of the presence of comorbidities falsely believed to be absolute contraindications to their use. To promote proper use of β-blockers in this article, we review the clinical pharmacology of β-blockers, the evidence of the beneficial effects of these drugs in heart failure with reduced ejection fraction, and the current guidelines for their use in clinical practice and in the presence of comorbidities (e.g., pulmonary disease, diabetes, atrial fibrillation, peripheral arterial disease, etc.). It is hoped that the practical approach discussed in this review will allow for a proper diffusion of knowledge about the correct use of β-blockers and the drug-disease interactions to achieve their increased use and titration, as well as for the selection of a specific agent with a view to a properly tailored approach for HFrEF patients.

## 1. Introduction

Heart failure (HF) is the leading cardiovascular disease, affecting approximately 26 million people worldwide [1,2]. Fortunately, in the last 30 years, the knowledge of the pathophysiology of this clinical syndrome has developed considerably, leading to the identification of drugs which can significantly improve the prognosis of these patients, called disease-modifying drugs [3,4,5].

Among them, β-blockers are indicated by international guidelines as the cornerstone of therapy for patients with HF with reduced ejection fraction (HFrEF) [6,7,8]. The optimal use of β-blockers has been shown to improve symptoms, reduce hospitalizations, induce left ventricular reverse remodelling, and increase survival in HFrEF patients [9,10]. Despite the proven benefit of β-blockers in chronic HF, they are often underutilized in current clinical practice [11,12]. There are a lot of well written reviews on the use of β-blockers in HFrEF; however, they only partially analyze the clinical aspects regarding this matter. To allow a proper diffusion of knowledge about the correct use of β-blockers, in this review we analyze the rationale and scientific evidence for the use of this drug in HFrEF patients with and without comorbidities, as well as provide practical tips for clinical cardiologists about the use of these drugs in the management of these patients.

## 2. Use of β-Blockers in HFrEF: Pathophysiology and Clinical Pharmacology

After a myocardial insult—whether acute (e.g., myocardial infarction or myocarditis) or chronic (e.g., hypertension or mitral valve insufficiency), which results in left ventricular dysfunction—the renin–angiotensin–aldosterone system and the sympathetic nervous system (SNS) are hyperactivated [13,14]. Persistent activation of the SNS in patients with HFrEF is evidenced by increased plasma levels of epinephrine and norepinephrine [15] and an increased spillover of the latter from sympathetic nerve endings into the bloodstream [16]. This increase in the catecholamines release leads to chronic and persistent stimulation of myocardial β-receptors, with consequent dysfunction and harmful repercussions for the failing heart [17,18].

Cardiac β-receptor dysfunction in HFrEF is characterized by a reduced β1-receptor density and by the uncoupling of β1- and β2-receptors from the membrane G proteins, resulting in their functional desensitization [19]. This mechanism is mediated by increased G protein-coupled receptor kinase 2 activity, resulting in reduced cardiac β-receptor density and reactivity, with consequent reduced cardiac inotropic reserve [20,21]. In addition, catecholamines themselves are cardiotoxic, contributing to myocardial damage (Table 1) [22,23].

Clinical consequences of these processes consist of reduced systolic function and left ventricular ejection fraction, acceleration of the left ventricular remodelling process, and the appearance of life-threatening ventricular arrhythmias [24].

Chronic β-blocker therapy results in direct antagonism of the cardiotoxic effects of catecholamines [25], upregulation of myocardial β-receptors with the restoration of their function and consequent increase in the inotropic reserve of the heart, suppression of elevated cardiotoxic neurohormonal systems (the renin–angiotensin–aldosterone and endothelin systems) [26], and prolongation of the diastolic phase with improvement of coronary flow [27].

All this action leads to improvements in the structure and function of the left ventricle (reverse remodelling). Other beneficial activities in patients with HFrEF include reducing heart rate [28] and blood pressure, reducing the burden of atrial and ventricular arrhythmias [29], and anti-ischaemic effects [30]. Moreover, β-blockers improve the contractility of viable but noncontractile myocardium in patients with ischaemic (hibernating myocardium) [31] and non-ischaemic (stunning myocardium) HFrEF [32].

β-blockers can be broadly classified [33] into:

(1) Nonselective β-blockers with similar β1 and β2 activity (none of the β-blockers belonging to this class is indicated for HFrEF);
(2) β1-selective with a higher affinity for β1-adrenoreceptors (metoprolol, bisoprolol, and nebivolol), preferred in patients with chronic obstructive pulmonary disease or mild asthma (nebivolol also facilitates nitric oxide release and is preferred in patients with arterial hypertension);
(3) β-blockers with additional α-1-adrenoreceptor antagonism and consequent peripheral vasodilation (carvedilol), preferred in patients with hypertension or documented higher peripheral vascular resistance.

β-blockers can also be subclassified as lipophilic or hydrophilic. Lipophilic drugs undergo rapid gastrointestinal absorption and extensive hepatic metabolism, resulting in low bioavailability and quick elimination. Hydrophilic drugs have longer half-lives but may accumulate in the presence of renal insufficiency. All β-blockers approved for patients with HFrEF are lipophilic and, therefore, do not require correction in patients with reduced renal function. Even so, dose adjustment and slow uptitration are needed in patients with severe hepatic impairment (i.e., Child–Pugh index > 10).

## 3. Evidence Supporting the Use of β-Blockers in HFrEF

The activation of the SNS is one of the significant pathophysiological abnormalities in HFrEF patients. Since the 1970s, it has been known that patients with HFrEF have higher plasmatic norepinephrine levels and that prognosis is directly related to catecholamine plasma levels. However, the first multicenter randomized trial was not conducted until the early 1990s, and carvedilol was approved for the treatment of HFrEF only in 1997. The reason for such a slow acceptance of the use of β-blocker therapy for HFrEF was the transient negative inotropic effect of the β-blockade and the subsequent risk of decompensation in patients with HFrEF.

The favorable outcome of the carvedilol trial resulted in a surge in research on the effects of β-blockade in HFrEF, including several randomized controlled trials of seminal importance (Table 2).

Metoprolol: The Metoprolol in Dilatated Cardiomyopathy (MDC) trial enrolled 383 patients with idiopathic dilated cardiomyopathy with ejection fraction (EF) below 40%, assigned to a metoprolol or placebo group. Patients in the metoprolol group had 34% (95% CI −6 to 62%, *p* = 0.058) fewer primary endpoints than the placebo group [34]. In addition, metoprolol was related to an increase in the ejection fraction (0.13 vs. 0.06, *p* < 0.0001) and exercise time (*p* = 0.046) from baseline to 12 months with respect to placebo. In the double-blind, randomized control trial Metoprolol CR/XR Randomized Intervention Trial in Heart Failure (MERIT-HF trial), metoprolol was compared with placebo in 3991 patients with HF and EF < 40% [35]. The study was stopped early on the recommendation of the independent safety committee because all-cause mortality was lower in the metoprolol CR/XL group than in the placebo group (relative risk: 0.66 (95% CI 0.53–0.81); *p* = 0.00009 or, adjusted for interim analyses, *p* = 0.0062). There were fewer sudden deaths in the metoprolol CR/XL group than in the placebo group (relative risk 0.59 (95% CI 0.45–0.78); *p* = 0.0002) and deaths from worsening heart failure (relative risk 0.51 (95% CI 0.33–0.79); *p* = 0.0023). Finally, in the trial Carvedilol or Metoprolol European Trial (COMET), 3029 patients with HF with EF < 35%, receiving angiotensin-converting enzyme inhibitors and diuretics, were randomly assigned to receive either carvedilol (25 mg twice daily) or metoprolol (50 mg twice daily) [36]. The all-cause mortality was 34% for carvedilol and 40% for metoprolol (hazard ratio 0.83 (95% CI 0.74–0.93), *p* = 0.0017).

Carvedilol: The 1996 U.S. Carvedilol Heart Failure Study enrolled 1094 HF patients with FE < 35%; 696 patients were treated with carvedilol (target dose: 25–50 mg twice daily), 398 with placebo [37]. The study was stopped early because carvedilol therapy was accompanied by a 27% reduction in the risk of hospitalization for cardiovascular causes (19.6% vs. 14.1%, *p* = 0.036), as well as a 38% reduction in the combined risk of hospitalization or death (24.6% vs. 15.8%, *p* < 0.001). In the randomized multicenter trial Carvedilol Post-Infarct Survival Control in Left Ventricular Dysfunction (CAPRICORN), the effects of carvedilol on morbidity and mortality in patients with prior myocardial infarction and EF < 40% were evaluated. The trial enrolled 1959 patients, randomly assigned to receive carvedilol (*n* = 975) or placebo (*n* = 984). The study demonstrated reduced cardiovascular and non-cardiovascular mortality in patients in the carvedilol group (12% vs. 15%, hazard ratio 0.77 (95% CI 0.60–098), *p* = 0.03) [38].

In the Carvedilol Prospective Randomized Cumulative Survival (COPERNICUS) study, 2289 patients with advanced HF and EF < 25% were randomized to receive carvedilol or placebo. After an average follow-up period of 10.4 months, mortality was reduced by 34% in the carvedilol group [39]. Carvedilol also reduced the number of days spent in hospital by 27% for any cause and by 40% for heart failure. Patients in the carvedilol group felt better and were less likely to have a severe adverse event related to HF.

Bisoprolol: In the trial Cardiac Insufficiency Bisoprolol Study I (CIBIS-I), 641 patients with HF and EF < 40% were randomly assigned to bisoprolol or placebo groups [40]. The observed difference in mortality between groups did not reach statistical significance (relative risk, 0.80; (95% CI 0.56–1.15), *p* = 0.22); however, bisoprolol significantly improved the New York Heart Association (NYHA) class (*p* = 0.04). In the Cardiac Insufficiency Bisoprolol Study II (CIBIS-II), 2647 patients with symptomatic HF (NYHA class III–IV) and EF < 35% were randomly assigned to receive bisoprolol or placebo [41]. The trial was stopped after the second interim analysis because bisoprolol showed a significant mortality benefit. All-cause mortality was significantly lower with bisoprolol than placebo (11.8% vs. 17.3%, hazard ratio of 0.66 (95% CI 0.54–0.81) *p* < 0.0001); there were also significantly fewer sudden deaths among patients on bisoprolol than in those on placebo (3.6% vs. 83 6.3%, hazard ratio of 0.56 (95% CI 0.39–0.80), *p* = 0.0011).

Nebivolol: Nebivolol is a selective antagonist of the β1-receptor. The drug also stimulates β3-receptors, inducing nitric oxide production and vasodilation. In the trial Study of the Effects of Nebivolol Intervention on Outcomes and Rehospitalization in Seniors with heart failure (SENIORS), over 2000 patients aged over 70 years and with EF < 35% were included in the study [42]. The trial showed a significant reduction in the composite endpoint of all-cause mortality and heart failure-related hospitalizations (31.1% vs. 35.3%, hazard ratio 0.86, (95% CI 0.74–0.99), *p* = 0.039]. However, the all-cause mortality decrease did not reach statistical significance.

Given these results, nebivolol was not included among the β-blockers indicated for the treatment of HFrEF in the American and Canadian guidelines. In contrast, it was included in the European guidelines with the note that it has not been shown to reduce cardiovascular or all-cause mortality in patients with HF.

## 4. Optimizing the Use of β-Blockers in HFrEF: A Practical Approach

**Figure 1:**
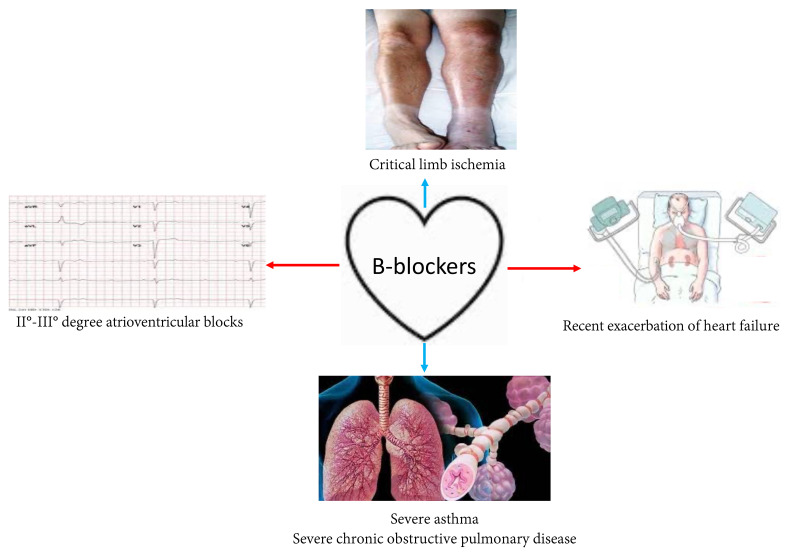
Cardiological (red arrow) and non-cardiological (blue arrow) contraindication to β-blockers therapy.

The prognostic benefits of β-blockers in HFrEF are such that they are included among the “fantastic four” drugs [43] used for the treatment of HFrEF; therefore, their prescription should always be considered in all patients with HFrEF. To obtain the maximum prognostic benefit from this class of drugs, adequate titration is required; therefore, a doubling of the dose every 2–3 weeks is necessary [5]. Careful evaluation of contraindications to their use (Figure 1) or factors that limit their uptitration should be performed. In clinical practice, the main factors that may limit the uptitration of β-blockers are:

(1) Peripheral congestion: β-Blockers should not be initiated in patients with moderate to severe fluid retention [44]. Because HFrEF is a progressive disease, it is likely that, during its course, many patients will develop signs and symptoms related to fluid retention. The initial approach to these patients is based on fluid management, often increasing the dose or adding a second diuretic (sequential nephron blockade) [45]. In the presence of congestion, initiation of therapy or increase in β-blocker dosage should be deferred until euvolemia is achieved [46]. In general, discontinuation or dose reduction of a β-blocker is not indicated in the presence of congestion unless it is associated with hypoperfusion (in case of a cold and wet patient).
(2) Asymptomatic hypotension: This is common in patients with HFrEF and is not a contraindication to β-blocker therapy [47]. It is essential to consider whether hypotension is caused by an inadequate preload related to aggressive use of diuretics or vasodilators [48]. In such cases, it may be necessary to reduce or suspend these therapies.
(3) Symptomatic bradycardia: β-Blockers may be used in patients with asymptomatic, mild bradycardia, particularly when the heart rate increases with exercise [49]. The possibility of drug interactions that may lower the heart rate (e.g., digoxin and amiodarone) should also be considered [50]. Given the substantial benefits of β-blockers in HFrEF, asymptomatic bradycardia during β-blocker therapy is not a reason to discontinue it, and cardiac pacing should be considered on an individual basis [51].

## 5. Use of β-Blockers in Patients with Heart Failure and Comorbidities: A Practical Approach

Comorbidities are highly prevalent in patients with HFrEF with a significant impact on disease progression and long-term prognosis [52]. Recent evidence shows that, in patients with HFrEF and multiple comorbidities, β-blockers are still able to achieve favorable prognostic effects [53]. Therefore, the optimization of β-blockers therapy and the appropriate selection of the best suited β-blocker (Table 3) is of paramount importance in this specific subgroup of patients.

### 5.1. Asthma and Chronic Obstructive Pulmonary Disease (COPD)

In about 30% of cases patients with HFrEF have an associated respiratory morbidity such as asthma or COPD [54]. The presence of these conditions should not limit the use of β-blockers, but it does influence the choice of the β-blocker type. COPD adversely affects the prognosis of patients with HFrEF, mainly in terms of recurrent hospitalizations, because of both the rapid impairment of haemodynamic balance due to COPD exacerbations [55] and the underuse of β-blockers in these patients [56]. The prognostic significance of β-blocker administration is independent of the presence of COPD at baseline, and reaching the maximum tolerated dose has significant prognostic significance in this context as well [57]. In addition, β1-selective agents (bisoprolol, metoprolol, and nebivolol) are preferred in patients with COPD because these drugs do not impact the clinical effects of β-agonists and they are not responsible for the worsening of respiratory symptoms or the of worsening FEV1 (i.e., the volume that has been exhaled at the end of the first second of forced expiration during spirometry), compared with placebo [58].

Even asthma does not represent an absolute contraindication to the use of β-blockers; in fact, in a population-based nested case-control study, it was shown that cardioselective β-blockers prescribed to people with asthma and HF were not associated with a significantly increased risk of moderate or severe asthma exacerbations and potentially could be used more widely when strongly indicated [59]. In addition, a network meta-analysis of randomized controlled trials showed that oral bisoprolol treatment was associated with a relatively lower incidence of asthma attacks than in the placebo group (relative risk 0.46 (95% CI 0.02–11.65)) [60]. In conclusion, the literature allows us to affirm that β1-blockers, particularly bisoprolol, are useful and safe in patients with HFrEF and COPD or asthma and represent the drug of choice in these patients.

### 5.2. Diabetes Mellitus (DM)

The use of β-blockers in patients with HFrEF and diabetes has historically been controversial [61]. The main reason for concern is the negative effect of β-blockers on glucose metabolism. Indeed, β-blockers might contribute to the development of hyperglycaemia by impairing the release of insulin from pancreatic β-cells [62]. In addition, β-blockers may mask the catecholamine-mediated symptoms of hypoglycaemia [63]. An analysis of six pivotal β-blocker trials, including 3230 patients with diabetes, showed that β-blockers significantly reduced mortality in individuals with (relative risk: 0.84 (95% CI 0.73–0.91)) and without (relative risk: 0.72 (95% CI 0.65–0.79)) diabetes [64]. Furthermore, concerns about worsening glycaemic control in patients with type 2 DM treated with β-blockers seem unfounded; in a recent study, in which 125 diabetic patients were enrolled, the use of carvedilol or bisoprolol did not worsen glycaemic control [65]. Therefore, the use of β-blockers should not be avoided in patients with HFrEF and diabetes. Carvedilol and bisoprolol should be used preferentially because they do not adversely affect the patients’ glycaemic profile.

### 5.3. Atrial Fibrillation (AF)

Atrial fibrillation represents the main supraventricular arrhythmia in patients with HFrEF, with a prevalence ranging from 20% to 40%, depending on the severity of the disease [66]. Recent studies have examined the prognostic difference of β-blocker therapy in patients with HFrEF based on heart rhythm. Kotecha et al. [67] reported no benefit of β-blockers regarding hospitalizations and mortality in the subgroup of HFrEF patients with AF (hazard ratio 0.97, (95% CI 0.83–1.14); *p* = 0.73). However, this study was a retrospective meta-analysis using somewhat older studies. In contrast to the above meta-analysis, the AF-CHF trial sub-study [68] showed an association between β-blocker treatment and reduced all-cause mortality (HR 0.72, 95% CI 0.54–0.94; *p* = 0.018), but not hospitalizations (HR 0.88; 95% CI 0.71–1.10; *p* = 0.223), even in patients with atrial fibrillation.

These data, like real-world data, document a prognostic advantage of β-blockers in all patients with HFrEF regardless of the presence of atrial fibrillation. Another important question is to what level of mean ventricular rate the patient with HFrEF and atrial fibrillation should be taken. The European Society of Cardiology guidelines state that the optimal resting heart rate in patients with AF and HF is unknown but may be between 60 and 100 beats per minute [69]. Randomized clinical trials that may clarify the optimal resting ventricular rate in patients with HFrEF and atrial fibrillation are pending, but it is the authors’ opinion that excessive rate control, associated with increased pauses, poses a risk in such patients. The use of β-blockers in these patients should therefore not be aimed at reaching target doses but at achieving a mean ventricular frequency of 70–80 beats/min to avoid prognostically unfavorable effects.

### 5.4. Peripheral Artery Disease

β-Blockers have been considered rather contraindicated in patients with peripheral artery disease and concomitant intermittent claudication. The decrease in cardiac output and the hypothesized blockade of β2-receptors (implicated in skeletal muscle vasodilation) are the main impacts of β-blockers invoked to support the cautious use of these drugs in patients with HFrEF and concomitant peripheral artery disease [70,71]. However, these pathophysiological assumptions are not supported by solid evidence but only by sporadic case reports and uncontrolled observations of worsening intermittent claudication and vasospastic phenomena associated with β-blocker use [72,73]. In addition, a meta-analysis by Cochrane collaboration (an international network of researchers belonging to this independent, not-for-profit organization) shows that, currently, no evidence suggests that β-blockers adversely affect walking distance, calf blood flow, calf vascular resistance, or skin temperature in people with intermittent claudication [74].

In conclusion, therapy with β-blockers can be safely conducted in patients with peripheral artery disease, in cases of severe intermittent claudication preferring those with vasodilation activity, such as carvedilol and nebivolol.

## 6. Knowledge Gaps and Outstanding Research Questions

There is a significant knowledge gap regarding the use of β-blockers in various HFrEF entities: renal dysfunction affects at least one in five HFrEF patients and is an important adverse prognostic factor. Traditionally, these patients have been excluded from randomized clinical trials, although evidence is accumulating of the value of β-blockers in such patients [75]. HFrEF has a higher incidence in elderly patients; however, few trials have enrolled patients with age > 70 years.

The possible place of β-blockade in HF with preserved ejection fraction remains controversial: data to date from trials of β-blockers in these patients have been inconsistent, although a recent registry study suggested benefits from this approach [76]. Further studies in these patients are required.

Finally, the correct place of β-blockade in the broad armamentarium of drugs indicated in patients with HFrEF (which is likely to increase in the coming years) remains to be clarified.

## 7. Conclusions

β-Blockers are one of the four disease-modifying drug types that have the greatest impact on the long-term prognosis of patients with HFrEF; consequently, all international guidelines recommend the use of β-blockers as a first-line therapy for patients with HFrEF. However, to date, they are underused, mainly because of the misconception that hypotension and bradycardia may worsen the haemodynamic status of patients with HFrEF and because of the presence of comorbidities falsely believed to be absolute contraindications to their use. A diffusion of knowledge about the correct use of β-blockers in clinical practice and drug–disease interaction is necessary for their greater use and titration as well as for the choice of a specific agent in view of a correct tailored approach to HFrEF patients.

## Figures and Tables

**Table 1:** Proposed mechanisms of catecholamine cardiotoxicity.

Type of Mechanisms
Imbalance of myocardial oxygen supply/demand
Calcium overload, with subsequent phosphorylation of multiple Ca(2+)-cycling proteins
Increased oxidative stress due the increased formation of “aminochromes”
Coronary spasm
Depletion of energy stores
Increased mitochondrial permeability

**Table 2:** Summary of randomized control clinical trials in heart failure with reduced ejection fraction. LVEF: left ventricular ejection fraction, NYHA: New York Health Association.

Trial	Year	Type of β-Blockers	n° of Patients	Inclusion Criteria	Effects on Mortality
CIBIS	1994	Bisoprolol	641	LVEF < 40%, NYHA class III-V	No significant difference in mortality between the two groups
MERIT HF	1999	Metoprolol	3991	LVEF < 40%, NYHA class II-IV	34% relative risk reduction in all-cause mortality
CIBIS II	1999	Bisoprolol	2647	LVEF < 35%, NYHA class III-IV	34% relative risk reduction in all-cause mortality
CAPRICORN	2001	Carvedilol	1959	Previous AMI and LVEF < 40%	23% relative risk reduction in all-cause mortality
COPERNICUS	2001	Carvedilol	2289	LVEF < 25% and NYHA class III-IV	31% relative risk reduction in all-cause mortality
COMET	2003	Metoprolol vs Carvedilolo	2309	LVEF < 35% and NYHA class II-IV	17% relative risk reduction in all-cause mortality in carvedilol group
SENIORS	2005	Nebivolol	2128	LVEF < 35%, NYHA class II-IV, age > 7o years	No significant difference in mortality between the two groups

**Table 3:** Choice of β-blockers according to clinical scenario.

Clinical Scenario	β-Blockers
Hypertension	Carvedilol, nebivolol
Asthma and Chronic Obstructive Pulmonary Disease	Bisoprolol, nebivolol
Diabetes mellitus	Carvedilol, bisoprolol
Atrial fibrillation	Metoprolol, bisoprolol
Peripheral Artery Disease	Carvedilol, nebivolol
Hypercholesterolemia	Carvedilol
Hyperthyroidism	Metoprolol
